# The orphan germinant receptor protein GerXAO (but not GerX3b) is essential for L-alanine induced germination in *Clostridium botulinum* Group II

**DOI:** 10.1038/s41598-018-25411-x

**Published:** 2018-05-04

**Authors:** Jason Brunt, Andrew T. Carter, Hannah V. Pye, Michael W. Peck

**Affiliations:** Gut Health and Food Safety, Quadram Institute, Norwich, UK

## Abstract

*Clostridium botulinum* is an anaerobic spore forming bacterium that produces the potent botulinum neurotoxin that causes a severe and fatal neuro-paralytic disease of humans and animals (botulism). *C*. *botulinum* Group II is a psychrotrophic saccharolytic bacterium that forms spores of moderate heat resistance and is a particular hazard in minimally heated chilled foods. Spore germination is a fundamental process that allows the spore to transition to a vegetative cell and typically involves a germinant receptor (GR) that responds to environmental signals. Analysis of *C*. *botulinum* Group II genomes shows they contain a single GR cluster (*gerX3b*), and an additional single *gerA* subunit (*gerXAO*). Spores of *C*. *botulinum* Group II strain Eklund 17B germinated in response to the addition of L-alanine, but did not germinate following the addition of exogenous Ca^2+^-DPA. Insertional inactivation experiments in this strain unexpectedly revealed that the orphan GR GerXAO is essential for L-alanine stimulated germination. GerX3bA and GerX3bC affected the germination rate but were unable to induce germination in the absence of GerXAO. No role could be identified for GerX3bB. This is the first study to identify the functional germination receptor of *C*. *botulinum* Group II.

## Introduction

*Clostridium botulinum* is the name given to a heterogeneous species that comprises four distinct groups of bacteria (*C*. *botulinum* Groups I to IV) that form the deadly botulinum neurotoxin^1–5^. The botulinum neurotoxin is the most potent toxin known, with a human lethal dose potentially being as little as 50 ng of neurotoxin^2,5^. There are a number of botulinum neurotoxin serotypes and subtypes^6–11^. Botulism in humans is most commonly associated with neurotoxin serotypes A, B or E, and more rarely with serotype F. There are three main types of human botulism. Foodborne botulism is a severe intoxication caused by consumption of food containing neurotoxin formed by *C*. *botulinum* during its growth in the food, whilst infant and wound botulism are both infections involving growth and neurotoxin formation in the body^1–5^. *C*. *botulinum* Groups I to IV are sufficiently distinct as to be considered separate species, and two of these Groups (*C*. *botulinum* Groups I and II) are associated with human botulism cases.

*C*. *botulinum* Group I is a mesophilic highly proteolytic bacterium that forms exceptionally heat resistant spores that are the target of the botulinum cook (121 °C/3 min) given to low acid canned foods^1–5^. Strains form up to three neurotoxins of serotype A, B and/or F. *C*. *sporogenes* is a close relative of *C*. *botulinum* Group I, and is often used as a surrogate in food safety tests^2,5,12^. However, recent genomic analysis indicates that *C*. *sporogenes* is not simply a non-toxigenic version of *C*. *botulinum* Group I. A majority (but not all) strains of *C*. *botulinum* Group I form botulinum neurotoxin, and while a fraction of *C*. *sporogenes* strains form serotype B neurotoxin, the majority of *C*. *sporogenes* strains do not form botulinum neurotoxin^8,13–16^. *C*. *botulinum* Group II is a psychrotrophic highly saccharolytic bacterium that forms spores of moderate heat resistance. Strains form a single neurotoxin of serotype B, E or F^1–5^. *C*. *botulinum* Group II is a concern for the continued safe production of minimally heated chilled foods^17–20^. The genomic diversity within *C*. *botulinum* Group II is greater than that within *C*. *botulinum* Group I/*C*. *sporogenes*, and *C*. *botulinum* Group II strains separate into two distantly related lineages. Lineage one contains the majority of serotype E strains, and these are strongly associated with the aquatic environment. Lineage two is dominated by serotype B strains, and also contains serotype F and occasional serotype E strains, and these strains are more frequently linked with terrestrial ecosystems. Interestingly, physiological differences have been reported between strains in the two lineages^5,8,21–30^. Spores formed by *C*. *botulinum* Groups I and II are widely distributed in the environment, and the prevention of human botulism relies on the identification and application of control measures that either prevent spores entering food or the body, or that prevent spore germination, cell multiplication and neurotoxin formation in food or the body. Spore germination is the key step in the transformation of the dormant resistant spore into multiplying vegetative cells that ultimately form botulinum neurotoxin. A greater understanding of spore germination can make an important contribution to microbiological food safety.

Spore germination has been widely studied in *Bacillus* species, and to a lesser extent in various species of *Clostridium*^31–34^. Spores germinate in response to nutrient germinants (e.g. amino acids) and to non-nutrient germinants (e.g. Ca^2+^: pyridine-2, 6-dicarboxylic acid (Ca^2+^-DPA)). Under favourable conditions spore dormancy is broken and germination ensues. In the environment, spore germination is generally initiated by a germinant receptor (GR) located in the spore inner membrane that responds to a nutrient germinant^35,36^. Following this initial step, the large depot of Ca^2+^-DPA within the spore is released, peptidoglycan in the spore cortex is hydrolysed, and core swelling and hydration is then followed by cell outgrowth. However, in *C*. *difficile* and *C*. *perfringens* spore cortex hydrolysis precedes Ca^2^^+^-DPA release. This general pattern of germination is followed in most species, with some notable differences for example in the germinants and how they are sensed, and how the cortex peptidoglycan is hydrolysed. Genes encoding four types of GR (*gerX1-4*) and 12 GR sub-types (that were designated by an additional letter) have been identified in *C*. *botulinum* Groups I to IV^37^. Genes encoding putative GR are well conserved within each *C*. *botulinum* Group, and are generally composed of three protein units GerA, GerB, and GerC. The position is similar in most other spore-forming bacteria, with germination requiring all three protein units to be present^34^. The genomic and physiological differences between the four *C*. *botulinum* Groups is also reflected in their germination apparatus and germination pathways^37^. Despite the importance of *C*. *botulinum* Groups I and II to both the food and pharmaceutical industry, our current understanding of nutrient induced germination and the role of the GR is limited, but is presently better understood in strains of *C*. *botulinum* Group I (and *C*. *sporogenes*) than in strains of *C*. *botulinum* Group II^37–39^.

Spores of Group I and Group II strains germinate in response to a variety of amino acids interacting with a GR^37,39–41^. Analysis of genome sequences has revealed the presence of three to five GR gene clusters in strains of *C*. *botulinum* Group I/*C*. *sporogenes*^37,39^. Detailed analysis revealed that amino acid induced germination in *C*. *botulinum* Group I strain ATCC 3502 required two GR (GerX1a and GerX1d) that were only functional when acting in synergy, while a function could not be identified for a third GR (GerX2b)^39^. In contrast, amino acid induced spore germination in *C*. *sporogenes* strain ATCC 15579 needed only a single GR (GerX1d), with two other GR contributing to the germination rate (GerX2c and GerX3a), and a fourth GR (GerX1a) having no discernible function^39^. The genetic complement of GR genes in *C*. *botulinum* Group II, however, is very different. Analysis of more than 150 *C*. *botulinum* Group II genome sequences has revealed the presence of only a single germinant receptor gene cluster (*gerX3b*) in each strain^37,40^. The *gerX3b* GR gene cluster has an unusual CA-B configuration that is different to the standard ABC configuration found in other *C*. *botulinum* Groups, as it has a bicistronic organization of *gerAC* and *gerB* genes orientated in opposite directions^37,40^. However, recently Clauwers *et al*. reported that this GR was not essential for amino acid induced spore germination in the *C*. *botulinum* Group II serotype E strain NCTC 11219^40^. Deletion of the *gerX3b* locus in this strain did not affect spore germination induced by a variety of amino acids, indicating that one or more unidentified GR are responsible for nutrient induced germination in strain NCTC 11219, and possibly also in other strains of *C*. *botulinum* Group II^40^. Strain NCTC 11219 belongs to *C*. *botulinum* Group II lineage one. The aim of the present study was to determine whether the GerX3b GR plays a major role in spore germination in strain Eklund 17B (that belongs to *C*. *botulinum* Group II lineage two) or whether the situation is similar to that reported by Clauwers *et al*.^40^ for strain NCTC 11219. We show that the GerX3b GR does not play a major role in spore germination in strain Eklund 17B, and have also identified a novel orphan germinant receptor, GerXAO, that is essential for amino acid induced germination.

## Materials and Methods

### Bacterial strains and growth conditions

*C*. *botulinum* Group II strain Eklund 17B (NRP) (serotype B) was grown anaerobically at 30 °C in tryptone-yeast-glucose medium (TYG). The *Escherichia coli* strain CA434^42^ was used for conjugal transfer and grown aerobically in Luria-Bertani medium (LB) at 37 °C. Where appropriate, growth media were supplemented with antibiotics at the following final concentrations; chloramphenicol 25 µg/ml, cycloserine 250 µg/ml, thiamphenicol 15 µg/ml, erythromycin 5 µg/ml and the chromogenic substrate 5-bromo-4-chloro-3-indolyl-β-D-galactopyranoside (X-Gal) 80 µg/ml. All bacterial media supplements were purchased from Sigma (Gillingham, UK). All culture and incubation experiments were performed in an anaerobic cabinet (Don Whitley, Shipley, UK) using an atmosphere of CO_2_: H_2_: N_2_ (5: 10: 85, v/v).

### Comparative genomics and phylogenetic analysis

For identification of *gerX* genes in the genomes of *C*. *botulinum* Group II strains, genome sequences were re-annotated using Prokka version 1.12^43^. Roary version 3.5.7^44^ was used for comparative genomics. Alignments were made with Clustal omega^45^ and MEGA version 6.0^46^ and used for generation of phylogenetic trees using the Neighbour Joining option. Figtree^47^ was used for annotation of phylogenetic trees. Conserved domains were identified using the Conserved Domain Database (NCBI)^48^.

### Spore preparation

*C*. *botulinum* strain Eklund 17B was maintained as described previously^41^. Spores were produced on a biphasic cooked meat medium plate (CMP). Robertson’s cooked meat broth (CMB) (20 ml) (Southern Group Laboratories) was inoculated with 0.1 ml of culture (grown overnight in TYG broth at 30 °C) and incubated for 24 h at 30 °C. CMP were prepared using homogenised CMB (200 ml) with 3 g of agar (Sigma) and 0.2 g of glucose (Fisher Scientific, UK). CMP were subsequently inoculated with 3 ml of the CMB culture and incubated at 30 °C for 7 days. Spores were harvested, cleaned, separated from cell debris by discontinuous density gradient (Urografin 370, Schering, Germany) centrifugation and stored at 2 °C in water, as described previously^41^. Phase contrast microscopy confirmed that the suspensions consisted of >95% phase-bright spores.

### Mutant generation

Genes encoding presumptive germination receptor (GR) sub-units (*gerX3bA*, *gerX3bB*, *gerX3bC)* in *C*. *botulinum* strain Eklund 17B were identified previously^37^, and *gerXAO* was identified in the present study. Mutants of *C*. *botulinum* strain Eklund 17B *gerX3b* subunits (*gerX3bA*; CB17B_RS15165, *gerX3bB*; CB17B_RS15170, *gerX3bC*; CB17B_RS15160, *gerXAO*; CB17B_RS12225) were generated using the Clostron system as previously described^39^. Briefly, target sites were identified using the Perutka method^49^ and mutants were generated as described^50^. Re-targeted introns were synthesised and ligated into the pMTL007C-E2 vector by DNA 2.0 (Menlo Park, USA). Retargeted intron plasmids were transformed into *E*. *coli* CA434. Confirmed (sequenced) plasmids were then transferred by conjugation into their respective clostridial host. For mutant complementation, plasmid pMTL83151 was used^51^. Primers bearing restriction sites compatible with pMTL83151 (*Bam*HI and *Nhe*I) were used to amplify the *gerX3b* GR sub-units and its 5′ noncoding region, covering the predicted putative promoter. The resulting PCR product was digested with *Bam*HI and *Nhe*I before being ligated into the pMTL83151 plasmid. Following confirmation by sequencing, complementation plasmids were transconjugated into their respective mutants using *E*. *coli* CA434 as described previously. Constructed mutants and plasmids utilised in this study are presented in Table S1. Primers used for verification of successful insertion events are listed in Table S2. PCR experiments were carried out using Phusion High-Fidelity PCR Master Mix with GC Buffer kit (Thermo Fisher). Plasmid isolation and PCR purification was performed using the Wizard *Plus* SV Minipreps DNA Purification System and the Wizard SV Gel and PCR Clean-Up System (Promega) respectively, as described in the provided Technical Manual. Chromosomal DNA isolation from suspected mutants were prepared as previously described^52^.

### Spore germination

The germinant L-alanine (50 mM) (Sigma) was prepared in phosphate buffer (100 mM, pH 7.0) with NaHCO_3_ (50 mM) and L-lactate (50 mM). Germinant solutions were filter sterilised (0.45-*µ*m syringe filter, Millipore, Bedford, MA, USA) and used within 1 h. Spore germination was also evaluated using Ca^2+^-DPA (60 mM, pH 7.4) in 20 mM Tris-HCl (pH 7.4) and NaHCO_3_ (50 mM). All spore suspensions were heat activated at 60 °C for 15 min, prior to the addition of potential germinants. Germination of spores at 30 °C was measured by a decrease in optical density (OD) at 600 nm every 5 min using a Bioscreen C analyser system (Labsystems, Basingstoke, UK). All germination experiments were performed in an anaerobic cabinet (Don Whitley) containing CO_2_: H_2_: N_2_ (5: 10: 85). Germination was expressed in terms of measured OD_600_ as a percentage of the initial OD_600_. To validate the OD_600_ measurements the proportion of germinated spores was visualised by the assessment of 200 spores in at least ten fields using phase-contrast microscopy. Typically, full germination was indicated when the OD_600_ fell to ~50–60% of its initial value. In some tests, a small fall in OD_600_ was observed (<10% of initial value). This was attributed to settling of spores in the Bioscreen wells, and was not accompanied by microscopic observation of spore germination. All experiments were performed in triplicate and the standard deviation calculated from three independent experiments. Statistical analysis of germination was completed using the two-tailed Student’s T-test with a significance level of 0.05.

### Data Availability

The datasets generated during and/or analysed during the current study are available from the corresponding author on reasonable request.

## Results and Discussion

**Figure 1:**
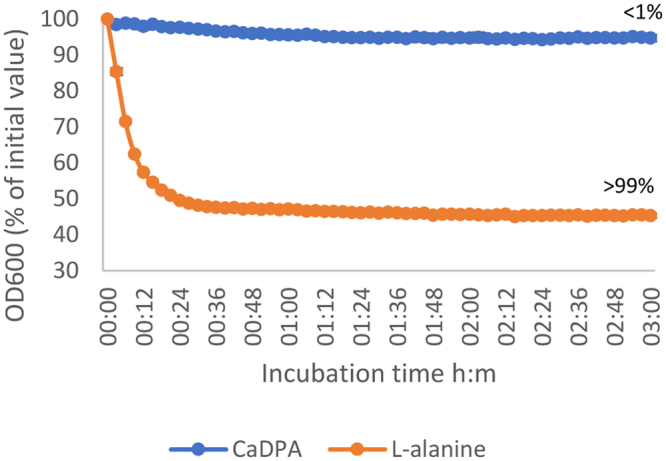
Effect of L-alanine (50 mM) and exogenous Ca^2+^-DPA (60 mM) on germination of *C*. *botulinum* Eklund 17B wild type spores. Data labels (right) refer to percentage germination observed by phase contrast microscopy at 3 hours. Error bars represent the standard deviation of 3 independent experiments.

The effect of two potential germinants, L-alanine and exogenous Ca^2+^-DPA, on the germination of *C*. *botulinum* strain Eklund 17B spores was evaluated (Fig. 1). In the presence of L-lactate (50 mM) and NaHCO_3_ (50 mM) at 30 °C, the addition at 50 mM of L-alanine initiated spore germination, as observed by a > 50% drop of initial OD_600_, after approximately 30 min (Fig. 1). Direct spore counts by phase contrast microscopy revealed a ~50% drop in OD_600_ correlated to >99% spore germination. Similarly, amino acid induced spore germination of other *C*. *botulinum* Group II strains has been previously reported^40,41^. As previously described, L-lactate was an essential co-germinant for L-alanine germination but did not stimulate germination on its own^41^. Similarly, although experiments were performed under anaerobic conditions, this was considered non-essential^41^. The mode of action of L-lactate on spore germination is unknown, but may include one of the following; (1) L-alanine and L-lactate interact before interacting with the GR, (2) L-lactate may directly affect the GR together or sequentially with L-alanine or (3) L-lactate affects yet unidentified proteins that are part of the germination pathway. The GR-independent germinant Ca^2+^-DPA (60 mM) is a non-nutrient germinant of some *Clostridium* and *Bacillus* species^34^, including *C*. *sporogenes* and *C*. *perfringens*^47,53,54^. However, exogenous Ca^2+^-DPA failed to induce spore germination in C. *botulinum* Eklund 17B, as determined by optical density measurement and phase contrast microscopy (Fig. 1). This observation is consistent with that made in a recent study with *C*. *botulinum* Group II strain NCTC 11219 where exogenous Ca^2+^-DPA also failed to induce spore germination^40^. Spores of strains in both *C*. *botulinum* Group II lineage one (strain NCTC 11219) and in lineage two (strain Eklund 17B) are therefore not germinated by exogenous Ca^2+^-DPA. It would be interesting to test the effect of exogenous Ca^2+^-DPA on strains in all four *C*. *botulinum* Groups. Spores of *Clostridium* and *Bacillus* species both contain a large amount of a 1:1 chelate of Ca^2+^ and pyridine-2, 6-dicarboxylic acid (DPA) within their core. In *Bacillus* and *C*. *sporogenes*, exogenous Ca^2+^-DPA activates the cortex lytic enzyme CwlJ to trigger spore germination^47,54^, whereas in *C*. *perfringens* Ca^2+^-DPA triggers germination through the GerK GR^53^. The failure of exogenous Ca^2+^-DPA to induce *C*. *botulinum* Eklund 17B spore germination in the present study (and strain NCTC 11219 previously) could be due to the absence of CwlJ, as there are no genes encoding CwlJ homologs in *C*. *botulinum* Group II genomes^37^. Moreover, *C*. *botulinum* Group II genomes do not contain the cortex lytic enzyme SleB found in most *Bacillus* and clostridia^34^ but instead possess the cortex lytic enzyme SleC as a zymogen. As with *C*. *botulinum* Eklund 17B, *C*. *difficile* spores do not germinate with the addition of Ca^2+^-DPA^55^, and their genomes lack genes encoding a homolog of CwlJ and also a ‘classical’ nutrient GR^55,56^ although they do possess a SleC similarly to *C*. *botulinum* Group II strains.

**Figure 2:**
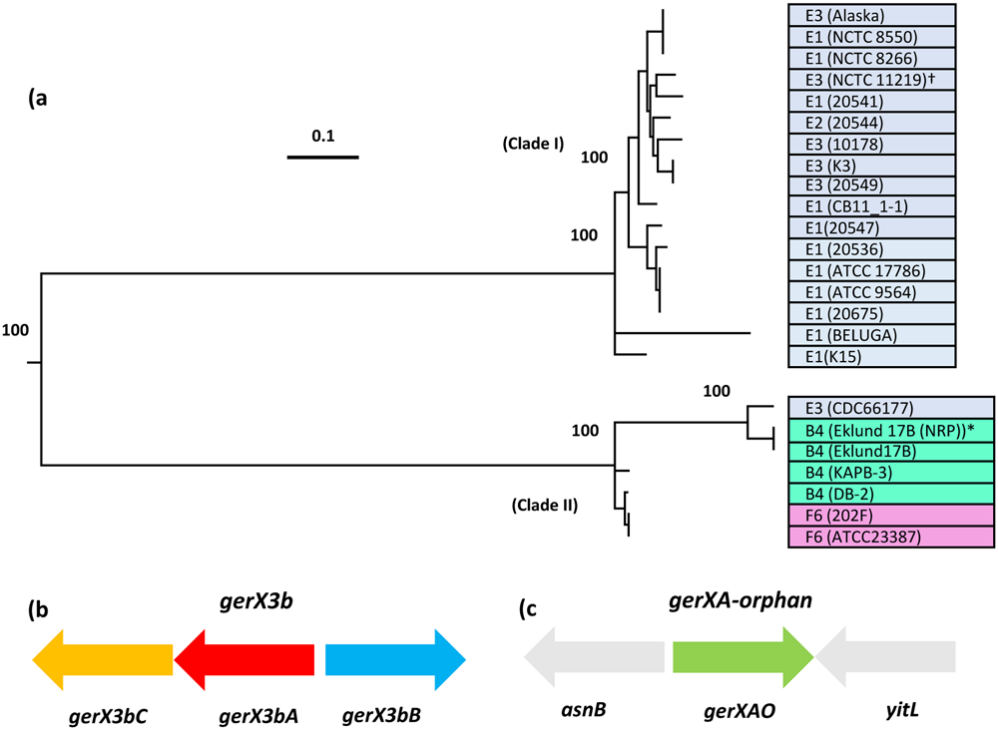
Relatedness of GerX3b receptor gene clusters in *Clostridium botulinum* Group II and genetic organization of the *gerX* subtypes. (**a**) Relatedness of *gerX3b* types in *C*. *botulinum* Group II (toxin types shown in coloured boxes). *gerX* associated proteins used for comparisons; *gerX3bA* (Locus tag; CB17B_RS15165), *gerX3bB* (Locus tag; CB17B_RS15170), *gerX3bC* (Locus tag; CB17B_RS15160) and *gerXAO* (Locus tag; CB17B_RS12225). *Represents strain *C*. *botulinum* Eklund 17B (NRP) (Accession number; FR745875) used in this study. ^†^Represents strain *C*. *botulinum* NCTC 11219 for comparison. Clade I consist of strains forming neurotoxin type E, and Clade II includes strains forming type neurotoxin types B, E or F. Strains used for comparison of the *gerX3b* types see Brunt *et al*.^37^. The phylogenetic tree is based on single nucleotide polymorphisms (SNPs). Values shown at branches represent bootstrap values provided by ParSNP. (**b**) Genetic organization of the *gerX3b* gene cluster. (**c**) *gerXAO* is flanked by asparagine synthetase (*asnB*) and an uncharacterized S1 RNA binding domain protein (*yitL*).

In most species, spore germination is initiated by a germinant receptor (GR) located in the spore inner membrane. The *C*. *botulinum* Eklund 17B genome includes a *gerX3b* (*gerX3bA*, *gerX3bB*, *gerX3bC*) receptor (Fig. 2a,b), similar to those described in other *C*. *botulinum* Group II strains^37,40^. Based on the sequence of the entire germinant receptor cluster, strains of *C*. *botulinum* Group II can be separated into two clades (Fig. 2a) that directly align with the two lineages derived from whole genome analysis^8,28^. The *gerX3b* germinant receptor in strain Eklund 17B is located in clade two in Fig. 2a (while the *gerX3b* germinant receptor in strain NCTC 11219 studied by Clauwers^40^ is found in clade one). Clade one exclusively contains strains forming type E neurotoxin, while clade two comprises strains forming type B, E or F neurotoxin (Fig. 2a). The *gerX3b* receptor gene cluster has a bicistronic organization, with *gerAC* and *gerB* genes being orientated in the opposite direction (CA-B configuration) (Fig. 2b). Analysis of more than 150 *C*. *botulinum* Group II genome sequences also revealed the presence of a single *gerA* subunit (termed here *gerXAO*, Fig. 2c) in each strain, indicating its likely importance. This single *gerA* subunit was distantly located on the chromosome from the *gerX3b* receptor gene cluster. The *gerXAO* gene, in general, is flanked by an asparagine synthetase (*asnB*) and an uncharacterized S1 RNA binding domain (*yitL*) gene. The annotation of this single *gerA* subunit is somewhat inconsistent with annotations frequently describing it as encoding a germination protein (GerA spore germination protein), or more rarely a sporulation protein (Stage V sporulation protein AF (SpoVAF)). However, Conserved Domain analysis confirmed that the GerXAO protein belongs to the GerA superfamily (cl10605). BlastP analysis also revealed high similarity between orphan GerA gene products from *C*. *botulinum* Group II strains (90–100%) and those identified by sequence similarity as spore germination proteins from *C*. *perfringens* and *C*. *taenosporium* (61% and 90% respectively). These are also not associated with either an ABC- or CA-B-type cluster structure. Interestingly, peptide sequence alignment of the GerXAO gene product with the receptor-associated (CA-B) GerA revealed only ~27% similarity at the amino acid level.

**Figure 3:**
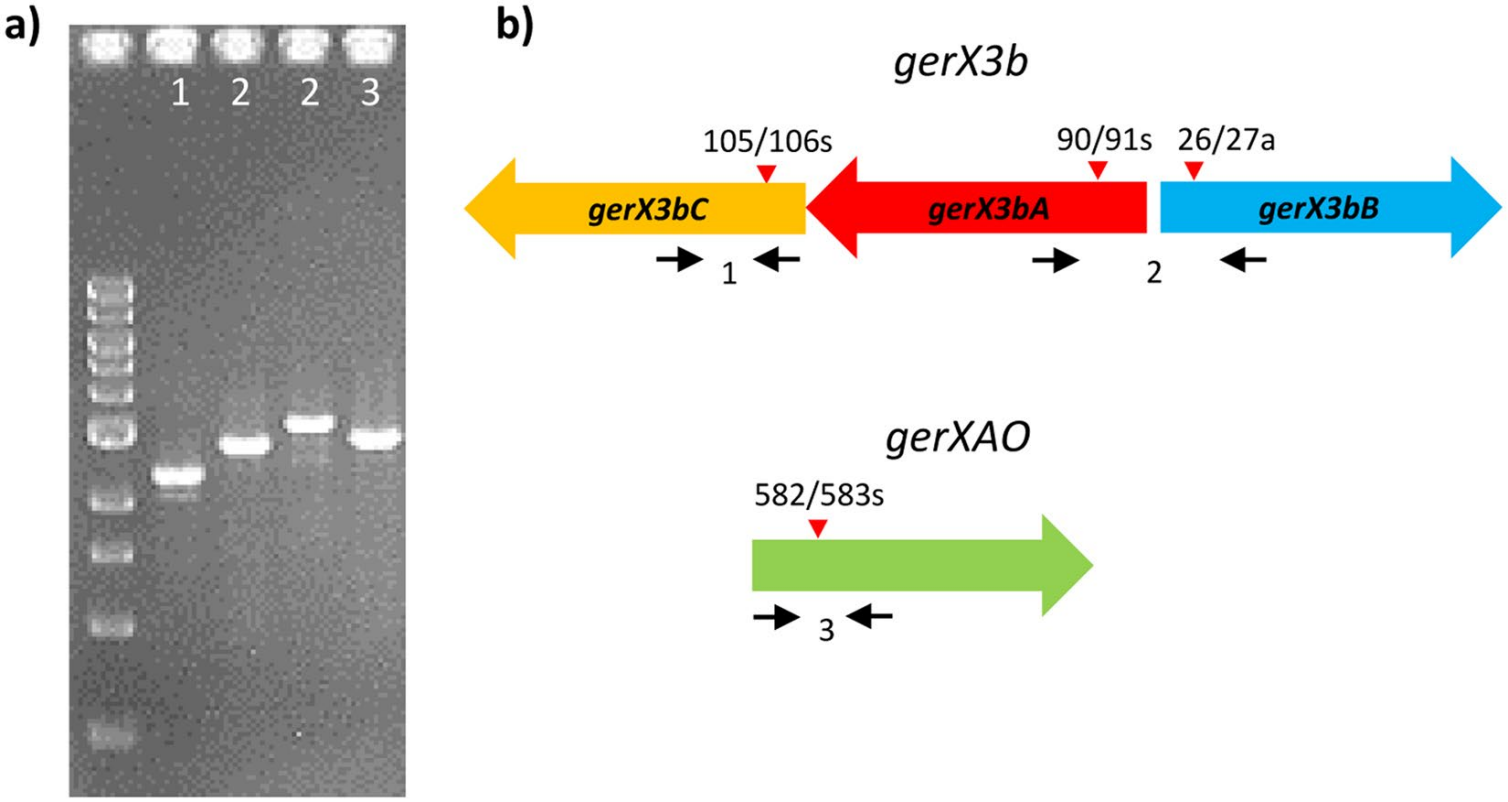
Confirmation of insertional mutagenesis. (**a**) PCR screens of *C*. *botulinum gerX* gene mutants confirming that the intron has successfully inserted into the target gene. Numbers represent primer sets used. Gel image has been cropped, full image available in supplementary figure (S1). (**b**) *C*. *botulinum* germination genes showing Clostron insertion sites (red arrows). Black arrows represent primer annealing sites. Primer set 1 (*gerC*-F/R) anneals to the target gene CB17B_RS15160 *gerX3bC* either side of the intron insertion site and confirms the intron is present in the target gene (expected band size of mutants ~2 kb). Primer set 2 (*gerAB*-F/R) anneals to the target gene CB17B_RS15165 *gerX3bA* and CB17B_RS15170 *gerX3bB* either side of the intron insertion sites and confirms the intron is present in the target gene. Primer set 3 (*gerAO*-F3/R3) anneals to the target gene CB17B_RS12225 *gerXOA* either side of the intron insertion sites and confirms the intron is present in the target gene.

**Figure 4:**
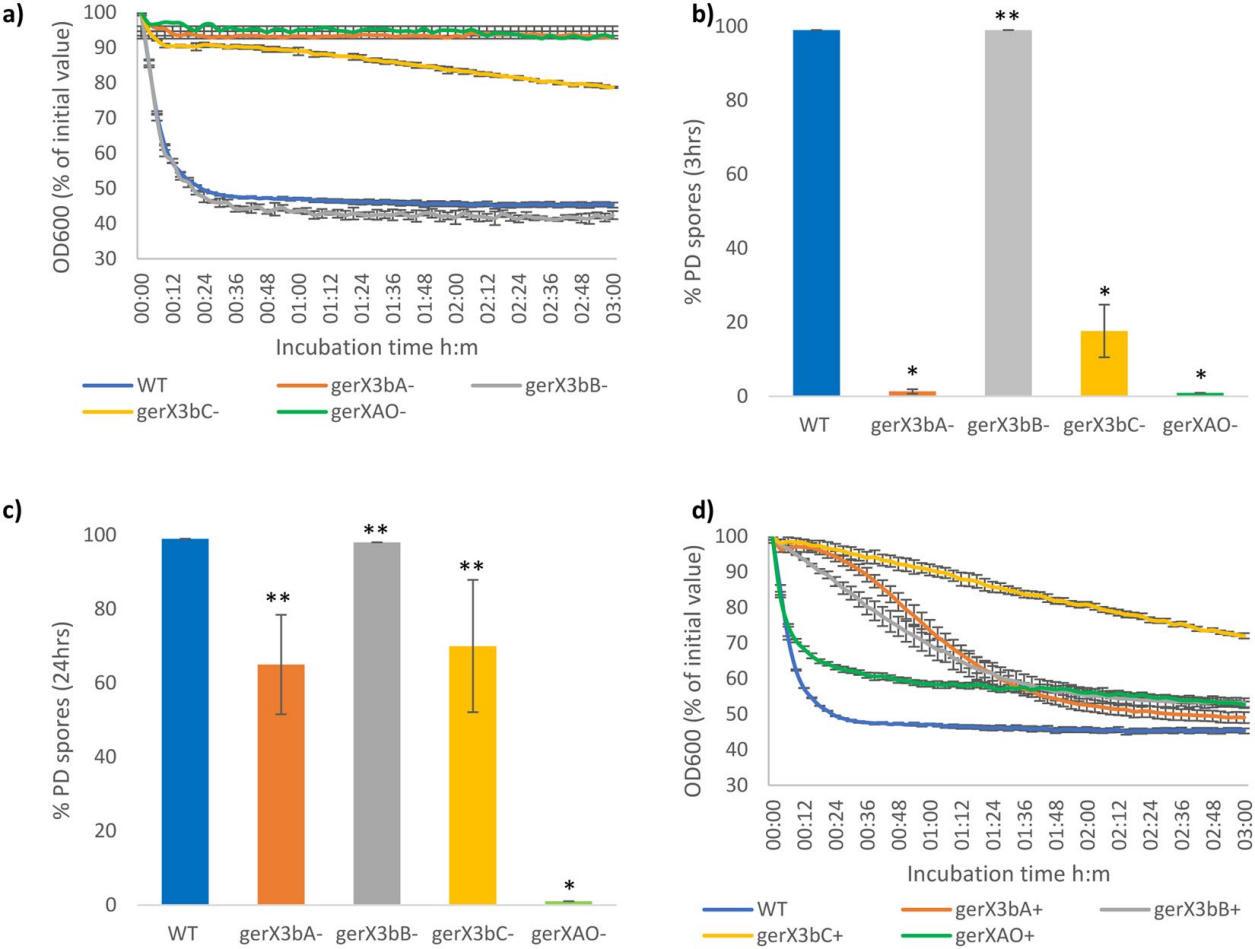
Effect of L-alanine (50 mM) on spore Germination of *C*. *botulinum* Eklund 17B *gerX3bA*^−^, *gerX3bB*^−^, *gerX3bC*^−^, *gerXAO*^−^ and wild type. (**a**) Kinetics of spore germination determined using fall in OD_600_. (**b**) Spore germination after 3 hours (percentage of phase dark spores as determined by phase contrast microscopy). (**c**) Spore germination after 24 hours (percentage of phase dark spores as determined by phase contrast microscopy). (**d**) Kinetics of spore germination of the complemented mutants determined using fall in OD_600_. Error bars represent the standard deviation of three independent experiments. *Student’s T-test with a significance level of 0.05. **Not significant at level of 0.05 compared to the wild type. ^+^complemented strains.

To characterise the functionality of the putative GR encoded by *gerX3b* (*gerX3bA*, *gerX3bB*, *gerX3bC)* in *C*. *botulinum* Eklund 17B, single insertional mutants of each gene were generated and confirmed by PCR and sequencing (Fig. 3). However, it should be noted that given the absence of formal characterisation of any *C*. *botulinum* gene promoters, mutation of *gerX3bA* may have a polar effect on *gerX3bC*. Mutation of *C*. *botulinum* Group II is challenging^40^, and several attempts were needed to isolate mutants for this strain. Spores generated from these mutants were analysed for L-alanine induced germination (Fig. 4). Mutants *gerX3bA*^**−**^ and *gerX3bC*^**−**^ failed to germinate (<10% fall in OD_600_) with L-alanine after 1 h of exposure (Fig. 4a). However, *gerX3bC*^**−**^ spores started to germinate slowly after 1 h (as determined by a fall in OD_600_). The OD_600_ of wild type spores decreased (∼50%) after ~30 min, representing efficient and complete spore germination (≥99%) as observed by phase contrast microscopy. Interestingly, the *gerX3bB*^**−**^ mutant exhibited comparable germination profiles to those of wild type spores indicating a lack of function in this stage of the germination process. This is in contrast to GerKB in *C*. *perfringens*, which is involved in spore viability and outgrowth^57^. The lack of phenotype for the Eklund 17B *gerX3bB*^**−**^ mutant is also surprising as the GerB protein in *Bacillus* is proposed to be responsible for germinant binding and influencing the GerC protein^58–60^. This apparent lack of function is additionally unexpected when the abundance of monocistronic *gerB* genes is considered, especially in the knowledge that *C*. *botulinum* Group II genomes are relatively stable with the potential for horizontal gene transfer considered low^8,28^. Further work is required, including examination of the orphan monocistronic *gerB* genes in *C*. *botulinum* Group I.

Phase contrast microscopy was used to determine the percentage of spores that had germinated (i.e. become phase dark) after 3 h and 24 h at 30 °C (Fig. 4b,c). Incubation with L-alanine for 3 h, resulted in negligible germination of mutant *gerX3bA*^−^ and limited germination of mutant *gerX3bC*^−^, but complete germination of the wild type and *gerX3bB*^−^ (Fig. 4b). Following incubation with L-alanine for 24 h, mutants *gerX3bA*^−^ and *gerX3bC*^−^ displayed germination approaching that of the wild type and *gerX3bB*^**−**^ (Fig. 4c). Complementation of *gerX3bA*^−^ and *gerX3bB*^**−**^ using plasmid pMTL83151 containing the *gerX3b* GR sub-units and their 5′ noncoding region restored the germination phenotype of *gerX3bA*^**−**^ and did not alter the wild type phenotype of *gerX3bB*^**−**^. Albeit, the rates of germination of these complemented mutants were not fully restored compared to that of the wild type (Fig. 4d). Complementation of *gerX3bC*^−^ did not restore the germination rate phenotype; this lack of successful complementation has been observed for many generated mutants in clostridia, but the reason is unclear^37,39,61,62^. However, in the absence of formal characterisation of any *C*. *botulinum* gene promoters, apart from BotR regulated ones in the neurotoxin gene cluster, we predicted that the promoter to be located in the intergenic region upstream of the *gerX3bA* gene and therefore the complementation of *gerX3bC*^**−**^ may have failed due to the lack of a native promoter. Furthermore, attempts at expressing the whole receptor failed so complementation of *gerX3bC*^**−**^ strain could not be assessed. The configuration of the bicistronic divergent *gerCA* and *gerB* gene cluster found in all *C*. *botulinum* Group II strains is comparable to the *gerK* locus reported in *C*. *perfringens* which comprises of a bicistronic *gerKA-gerKC* and monocistronic *gerKB* in the opposite orientation^53,63^. The similarity of the GR amino acid sequences for *C*. *perfringens* SM101 is approximately GerKA 63%, GerKB 36% and GerKC 46% when compared to those of *C*. *botulinum* Eklund 17B GerX3bA, GerX3bB, and GerX3bC, respectively. In *C*. *perfringens*, the GerKC protein is considered to be the main GR with GerAA and GerKB proteins playing a minor role in spore germination^53,63^. However, a recent report has now demonstrated that in the *C*. *botulinum* Group II strain NCTC 11219 GerX3b was not required for nutrient induced germination^40^. In this work, the entire GR cluster was deleted, and germination was similar in the wild type and mutant after 4 h/30 °C, as measured by number of heat resistant spores and DPA loss. Phase contrast microscopy additionally revealed that the rate of formation of phase dark spores was similar and virtually complete after 1 h/30 °C^40^. The findings made in the present study with strain Eklund 17B are consistent with the observation made with strain NCTC 11219 that the GerX3b receptor is not essential for spore germination in strains of *C*. *botulinum* Group II, and importantly now extend the finding to both major *C*. *botulinum* Group II lineages. We also demonstrate, for the first time, that in strain Eklund 17B two of the GR receptor proteins contribute to the germination rate and in their absence the germination rate is much slower with L-alanine compared to the wild type. The presence of *gerX3b* in the genome of all *C*. *botulinum* Group II strains examined to date^37,40^ indicates that the product of this gene cluster is likely to have an important biological role, for example it may respond to an as yet unknown nutrient/non-nutrient germinants, or perform a structural role for instance in the organisation of a germinosome^64^.

Given the findings presented here and those made by Clauwers *et al*.^40^ that the GerX3b receptor does not play a major role in spore germination in *C*. *botulinum* Group II, functional GR were now sought, and the potential role of GerXAO was assessed. Following insertional mutagenesis, spores were produced, and tested for L-alanine induced germination. Mutant *gerXAO*^**−**^ failed to germinate (<10% fall in OD_600_) with more than 99% of spores appearing phase bright after incubation for 24 h (Fig. 4). Additionally, complementation of *gerXAO*^**−**^ using plasmid pMTL83151 restored germination to similar levels of the wild type (≥99%). Thus, GerXAO plays a major role in spore germination in Eklund 17B.

**Figure 5:**
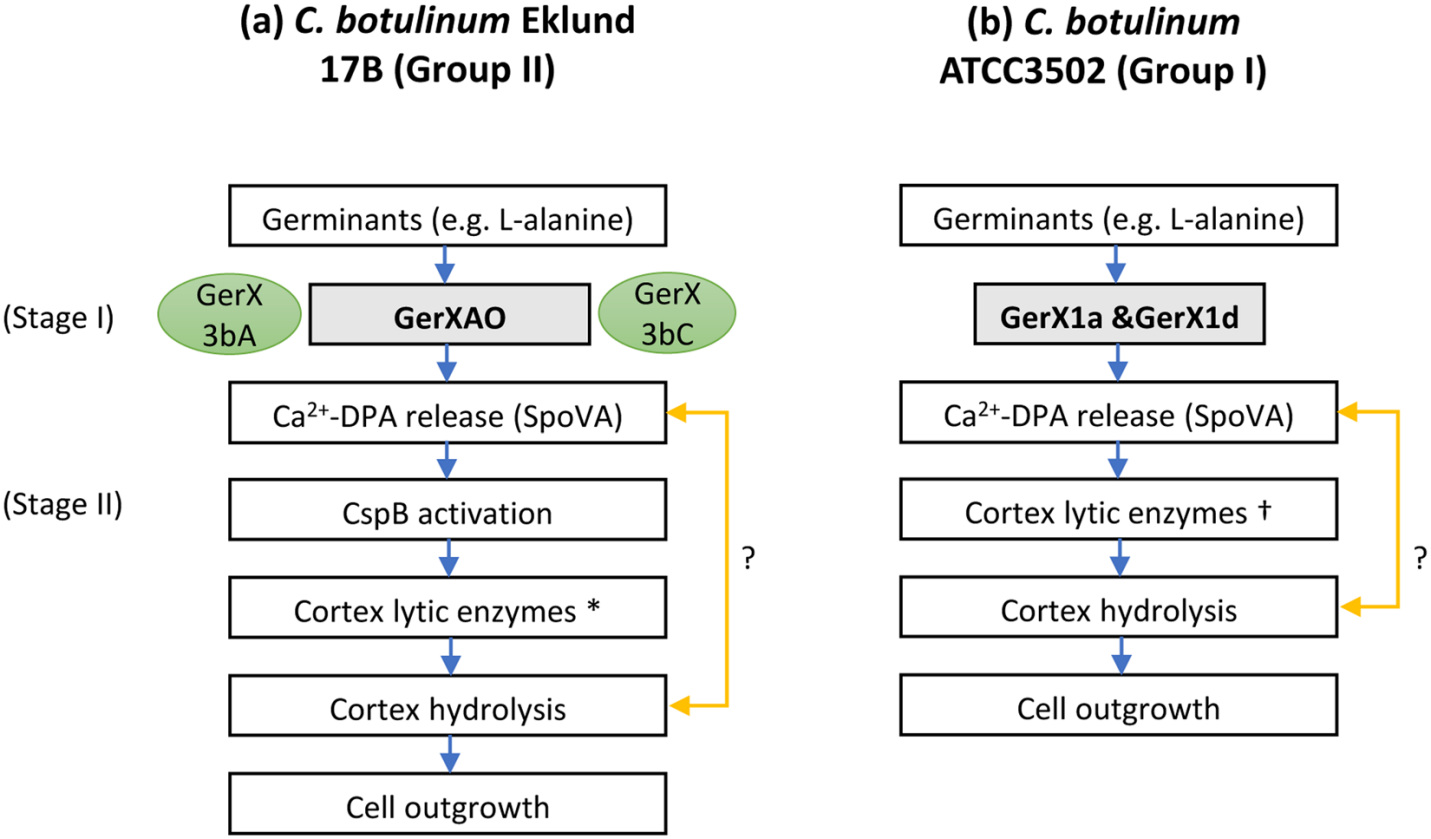
Comparison of the models proposed for the germination pathways of *C*. *botulinum* Group I and Group II. (**a**) Group II, with GerXAO (shaded grey) as the major germinant receptor and GerX3bA & GerX3bC (shaded green) involved in germination rate. Initially, L-alanine binds to the GerXAO receptor, followed by Ca^2+^-DPA release through the SpoVA channel (Stage I). In stage II, the cortex lytic enzyme SleC is activated by CspB, followed by cortex hydrolysis, membrane, and coat degradation, the resumption of metabolism and ultimately cell outgrowth. *Cortex lytic enzymes include SleC2 a/b and SleB2. (**b**) Germination pathway for *C*. *botulinum* strain ATCC3502 (Group I)^37,39^. This system involves the recognition of nutrient germinants by their cognate receptor GerX1a &GerX1d acting together, followed by Ca^2+^-DPA release through the proposed SpoVA channel (Stage I). In stage II, the CLEs CwlJ and SleB (†) are activated, followed by cortex hydrolysis, membrane and coat degradation, the recommencement of metabolism and eventually cell outgrowth. “?” indicates that the timing of the release of Ca^2+^-DPA is presently unknown in *C*. *botulinum* Group I and Group II and may be released following germinant binding or after cortex hydrolysis.

An orphan *gerA* gene is located in the genome of several species of *Clostridium*^32,65^, and is present in the genome of all of more than 150 strains of *C*. *botulinum* Group II examined in the present study, which indicates there is an evolutionary advantage to its retention as a functional gene. However, the orphan GerA present in *C*. *perfringens* played only a minor role in germination^53^. Tentative evidence in this study also revealed that sporulation efficiency appeared to be affected by mutation of *gerXAO*, as determined by phase contrast microscopy. Again, the reasons for this are unclear, we can only speculate that the loss of this receptor protein has an effect on an as yet unknown protein or signal that is involved in sporulation. Although this is the first report of an essential requirement for a monocistronic GerA in spore germination, function has been attributed to a monocistronic GerB which is involved in germinant specificity and rate in spores of *Bacillus megaterium* QM B1551^66^. Moreover, this GerB receptor subunit interacts with GerUA and GerUC to form a functional receptor. In *C*. *botulinum* Group II strain Eklund 17B, the rate of spore germination is more rapid when GerXAO interacts with GerX3bA and/or GerX3bC, than when either GerX3bA or GerX3bC are absent. However, further work is certainly warranted to understand the exact mechanism of the GerXAO receptor and to whether it responds to further germinants. Interestingly, the position in *C*. *botulinum* Group II strain Eklund 17B, where GerXAO brings about germination on its own with other proteins (GerX3bA and/or GerX3bC) increasing the rate, contrasts with the situation in most other spore-forming bacteria where germination involves a GR with three protein units (GerA, GerB, and GerC). A potential role for presently unidentified proteins that interact with GerXAO to bring about germination cannot also be excluded. Following the results presented here we now propose an updated version^37^ of the germination model for *C*. *botulinum* Group II strains (Fig. 5). Firstly, the nutrient germinant binds to the GerXAO receptor, followed by Ca^2+^-DPA release through the SpoVA channel (Stage I). In stage II, the cortex lytic enzyme SleC is activated by CspB, followed by cortex hydrolysis, membrane and coat degradation, the resumption of metabolism and ultimately cell outgrowth. Although, the order of these events still needs to be resolved in *C*. *botulinum* group II strains (Fig. 5)

In summary, this is the first report that successfully identifies the key component of the germination machinery of *C*. *botulinum* Group II strain Eklund 17B. The presence of the orphan gene encoding GerXAO in all *C*. *botulinum* Group II genomes so far examined strongly suggests that the germination model proposed in this work will stand for all Group II strains. The role of GerX3bB, a gene equally conserved but with no obvious function, remains un-resolved. However, it is clear that gene products of its neighbouring operon interact with that of *gerXAO* to increase the efficiency of the germination process.

## Electronic supplementary material

Table S1, S2 Fig. S1
