# Supplementary material for: The orphan germinant receptor protein GerXAO (but not GerX3b) is essential for L-alanine induced germination in Clostridium botulinum Group II
Source: Sci Rep. 2018 May 4;8:7060. doi: 10.1038/s41598-018-25411-x (PMC5935672; doi:10.1038/s41598-018-25411-x)

The orphan germinant receptor protein GerXAO (but not GerX3b) is essential for L-alanine induced germination in *Clostridium botulinum* Group II

Jason Brunt*, Andrew T. Carter, Hannah V. Pye, Michael W. Peck.

Table S1 Constructed mutants and plasmids utilised in this study

Strain	Genotype/information	Ref
Clostridium botulinum B str. Eklund 17B (NRP)	Group II strain, Type B neurotoxin	[1]
Eklund 17B(NRP) gerX3bA$^-$	Locus tag/insert site CB17B_RS15165-90|91s. Eklund 17B(NRP) *gerX3bA::erm*	This study
Eklund 17B(NRP) gerX3bB$^-$	Locus tag/insert site CB17B_RS15170-26|27a. Eklund 17B(NRP) *gerX3bB*::erm	This study
Eklund 17B(NRP) gerX3bC$^-$	Locus tag/insert site CB17B_RS15160-105|106s. Eklund 17B(NRP) *gerX3bC*::erm	This study
Eklund 17B(NRP) gerXAO$^-$	Locus tag/insert site CB17B_RS12225-582|583s. Eklund 17B(NRP) *gerXAO*::erm	This study
Eklund 17B(NRP) gerX3bA$^+$	Eklund 17B(NRP) *gerX3bA::erm /* pMTL83151-*gerX3aA*	This study
Eklund 17B(NRP) gerX3bB$^+$	Eklund 17B(NRP) *gerX3bB::erm /* pMTL83151-*gerX3aB*	This study
Eklund 17B(NRP) gerX3bC$^+$	Eklund 17B(NRP) *gerX3bC::erm /* pMTL83151-*gerX3aC*	This study
Eklund 17B(NRP) gerXAO$^+$	Eklund 17B(NRP) *gerXAO::erm /* pMTL83151-*gerXAO*	This study
Escherichia coli		
Top10	F- *mcrA* Δ(*mrr-hsd*RMS-*mcr*BC) Φ80*lac*ZΔM15 Δ *lac*X74 *rec*A1 *ara*D139 Δ(*araleu*)7697 *gal*U *gal*K *rps*L (StrR) *end*A1 *nup*G	(Invitrogen)
CA434	HB101 containing plasmid R702.	[2]
pMTL83151	pMTL83151 in CA434	This study
gerX3bA-pMTL83151	*gerX3bA*-pMTL83151 in CA434	This study
gerX3bB-pMTL83151	*gerX3bB*-pMTL83151 in CA434	This study
gerX3bC-pMTL83151	*gerX3bC*-pMTL83151 in CA434	This study
gerXAO- pMTL83151	*gerXAO*- pMTL83151 in CA434	This study
Plasmids		
pMTL007C-E2	Clostron plasmid, L1.LtrB intron with ermB RAM, ColE1 +tra, pCB102, Cm^R , constitutive intron expression under fdx promoter.	[3]
pMTL007C-E2::CB17B_RS15165-90|91s	Targets *gerX3bA* CB17B_RS15165, base 90 sense orientation	This study
pMTL007C-E2::CB17B_RS15170-26|27a	Targets *gerX3bB* CB17B_RS15170, base 26 antisense orientation	This study
pMTL007C-E2::CB17B_RS15160-105|106s	Targets *gerX3bC* CB17B_RS15160, base 105 sense orientation	This study
pMTL007C-E2::CB17B_RS12225-582|583s	Targets *gerXAO* CB17B_RS12225, base 582 sense orientation	This study
pMTL83151	*Clostridium-E. coli* shuttle plasmid; pCB102 replicon, ColE1 + tra, Cm^R	[3]

Genotype information; a = antisense orientation insertion site; s = sense orientation insertion site. Numbers i.e. 90|91 specify clostron target site.

1 Stringer, S. C. *et al.* Genomic and physiological variability within Group II (non-proteolytic) *Clostridium botulinum*. *BMC Genomics* **14**, 333, doi:10.1186/1471-2164-14-333 (2013).
2 Purdy, D. *et al.* Conjugative transfer of clostridial shuttle vectors from *Escherichia coli* to *Clostridium difficile* through circumvention of the restriction barrier. *Mol. Microbiol.* **46**, 439-452, doi:DOI 10.1046/j.1365-2958.2002.03134.x (2002).
3 Heap, J. T. *et al.* The ClosTron: Mutagenesis in *Clostridium* refined and streamlined. *J Microbiol Methods* **80**, 49-55, doi:10.1016/j.mimet.2009.10.018 (2010).

Table S2 Primers used for verification of successful insertion events

Primer	Sequence	Description
***gerAB*-F**	CCTCTAGTCGGAAATCCCTTTAC	Anneals to the target gene for
***gerAB*-R**	CCCAAGTATCTTGTTTGCCATATT	mutant confirmation in genes
		CB17B_RS15165 (*gerX3bA)* &
		CB17B_RS15170 (*gerX3bB)*
***gerC*-F**	AATCCTAAGTACTCTTCGTCACTGC	Anneals to the target gene for
***gerC*-R**	GGACTACCGGGATTCTTTATGTC	mutant confirmation in gene
		CB17B_RS15160 (*gerX3bC)*
**gerAO-F3**	CTGTGCTTTGTGGGCAAACA	Anneals to the target gene for
**gerAO-R3**	TGCAACGTCAGGTCTTTCTGT	mutant confirmation in gene
		CB17B_RS12225 (*gerXAO)*
**CB17B_RS15165gerAF**	CCTTTTGGATCCCATCTCTGATTGCCTCTAAA	Primers used to produce *gerX3bA*
**CB17B_RS15165gerAR**	CCTTTTGCTAGCGGCTAATTAATGTAGTTGCT	complement construct
**CB17B_RS15170gerBF**	CCTTTTGGATCCGTATCAACATCCCTATACTTTA	Primers used to produce *gerX3bB*
**CB17B_RS15170gerBR**	CCTTTTGCTAGCGAGTATTCTCAAAGTCCAAT	complement construct
**CB17B_RS15160gerCF**	CCTTTTGGATCCTGTTGATATATGCACAATCA	Primers used to produce *gerX3bC*
**CB17B_RS15160gerCR**	CCTTTTCTCGAGGTATAGCATCAAGAATAACA	complement construct
**CB17B_RS12225gerAOF**	CCTTTTGGATCCGCAATACCACACATAATGTT	Primers used to produce *gerXAO*
**CB17B_RS12225AOR**	CCTTTTGCTAGCGCTCAAGACAAAGAAGGAAC	complement construct
**SBF-seq**	GCGCGTGATTGCCAAGCA	Plasmid primers for confirmation
**ASC-seq**	TCTGTAAATTTCTTTCTATTCAGCACTGT	of the presence of the *gerX*
		construct in plasmid pMTL83151

Figure S1 Confirmation of insertional mutagenesis (uncropped version of gel in Figure 3). Three replicates of PCR products. Numbers represent primer sets used. See Figure 3.

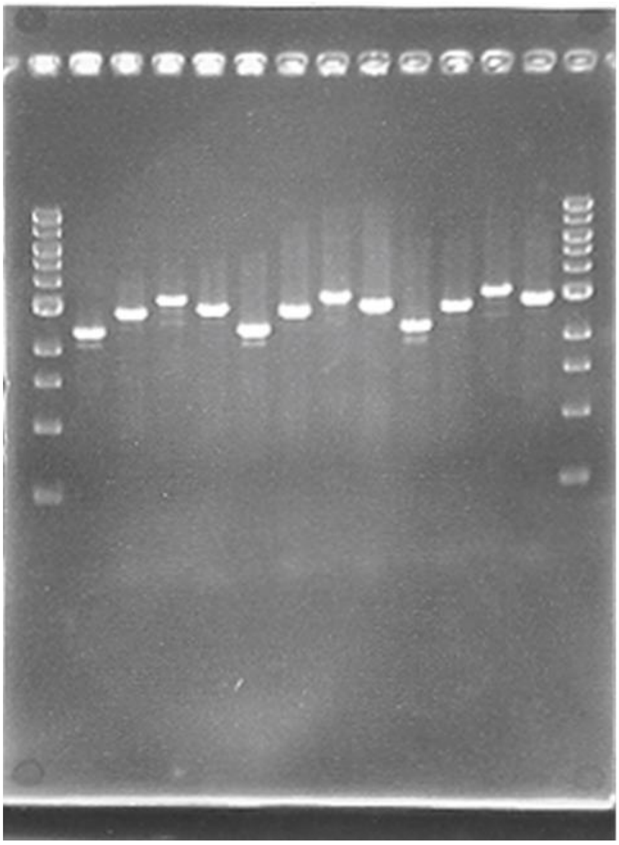

Supplement: Supplementary file 1 — Table S1, S2 Fig. S1 [file 41598_2018_25411_MOESM1_ESM.pdf]
